# Supplementary material for: Distribution and Associated Factors of Hepatic Iron—A Population-Based Imaging Study
Source: Metabolites. 2021 Dec 15;11(12):871. doi: 10.3390/metabo11120871 (PMC8705957; doi:10.3390/metabo11120871)
Supplement: Supplementary file 1 [file metabolites-11-00871-s001.zip › metabolites-1513974-supplementary.pdf]

Supplementary Material

Distribution of hepatic iron and associated factors – a population-based imaging study

Lisa Maier[1,2,3], Ricarda von Krüchten[4], Roberto Lorbeer[5,6], Jule Filler[1,2,3], Johanna Nattenmüller[4,7], Barbara Thorand[1,8], Wolfgang Koenig[6,9,10], Wolfgang Rathmann[8,11], Fabian Bamberg[4], Christopher L. Schlett[4], Annette Peters[1,2,6,8], Susanne Rospleszcz[1,2,6]

1 Institute of Epidemiology, Helmholtz Zentrum München, German Research Center for Environmental Health, Neuherberg, Germany

2 Institute for Medical Information Processing, Biometry and Epidemiology – IBE, LMU Munich, Munich, Germany

3 Pettenkofer School of Public Health, Munich, Germany

4 Department of Diagnostic and Interventional Radiology, Medical Center - University of Freiburg, Freiburg, Germany

5 Department of Radiology, University Hospital, LMU Munich, Munich, Germany

6 German Center for Cardiovascular Disease Research (DZHK), Munich, Germany

7 Department of Diagnostic and Interventional Radiology, University Hospital Heidelberg, Heidelberg, Germany

8 German Center for Diabetes Research (DZD), Neuherberg, Germany

9 German Heart Center Munich, Technical University Munich, Munich, Germany

10 Institute of Epidemiology and Medical Biometry, University of Ulm, Ulm, Germany

11 Institute of Biometrics and Epidemiology, German Diabetes Center, Duesseldorf, Germany

Table S1: Description of variable assessment.

Variable	Description
**Body composition**	
Body weight (kg)	Measured by calibrated steelyards or digital scales (SECA 635 or SECA 877 or SECA measuring station 285, Seca GmbH & Co, KG, Hamburg, Germany)
Height (cm)	Measured by by calibrated levelling bar (SECA 242, Seca GmbH & Co, KG, Hamburg, Germany)
BMI (kg/m^2)	Calculated by dividing weight in kilogram by squared height in meter
Waist circumference (cm)	Measured with an inelastic tape at the level midway between the lower rib margin and the iliac crest
Hip circumference (cm)	Measured with an inelastic tape at the level of maximal gluteal protrusion
Subcutaneous fat (L)	MRI measurement (3 Tesla): subcutaneous fat from the femoral head to the cardiac apex by a three-dimensional in/opposed-phase VIBE-Dixon sequence
Visceral fat (L)	MRI measurement (3 Tesla): visceral fat from the femoral head to the diaphragm by a three-dimensional in/opposed-phase VIBE-Dixon sequence
Total fat (L)	MRI measurement (3 Tesla): total fat from the femoral head to the cardiac apex by a three-dimensional in/opposed-phase VIBE-Dixon sequence
**Blood lipids**	
Total cholesterol (mg/dL)	Enzymatic, colorimetric CHOL Flex assay (Vista, Siemens or Cobas, Roche)
HDL-C (mg/dL)	Enzymatic, colorimetric HDLC Flex assay (Vista, Siemens or Cobas, Roche)
LDL-C (mg/dL)	Enzymatic, colorimetric LDLC Flex assay (Vista, Siemens or Cobas, Roche)
TG (mg/dL)	Enzymatic, colorimetric TRIG Flex assay (Vista, Siemens or Cobas, Roche)
**Markers of glucose metabolism**	
Fasting glucose (mg/dL)	UV test using enzymatic reference method with hexokinase (Vista, Siemens or Cobas, Roche)
Fasting insulin (mU/mL)	Elecsys Insulin immunoassay with two monoclonal antibodies (Vista, Siemens or Cobas, Roche)
HbA1c (%)	Cation-exchange high performance liquid chromatographic, photometric assay (VARIANT II TURBO Hemoglobin Testing System, Bio-Rad Laboratories Inc, Hercules, US)
2-hour insulin (µU/mL)	Serum insulin 2 hours post OGTT
2-hour glucose (mg/dL)	Serum glucose 2 hours post OGTT
Diabetes status<br>Categories:<br>*Normoglycemic*<br>*Prediabetes*<br><br>*Diabetes*	Established type 2 diabetes mellitus or results from OGTT<br><br>2-hour glucose <140 mg/dL and fasting glucose level <110 mg/dL<br>2-hour glucose between 140 and 200 mg/dL and/or an fasting glucose between 110 and 125 mg/dL, and normal 2-hour glucose<br>2-hour glucose >200 mg/dL and/or fasting glucose >125 mg/dL (1)

**Markers of renal function**	
Glomerular filtration rate (calculated from cystatin C)	Sex-specific calculation based on serum creatinine according to CKD-EPI
Glomerular filtration rate (calculated from cystatin C and serum creatinine)	Sex-specific calculation based on serum creatinine according to CKD-EPI
Uric acid (mg/dL)	Enzymatic colorimetric UA Flex assay (Vista, Siemens or Cobas, Roche)
Creatinine (mg/dL)	Kinetic colorimetric CREJ assay based on Jaffé method
Albumin (g/dL)	Bromocresol purple (BCP) dye-binding method with ALB Flex assay
Urine albumin (mg/L)	Immunonephelometry
Urine creatinine (g/L)	Kinetic colorimetric CREJ assay based on Jaffé method
Cystatin C (mg/L)	Particle-enhanced immunonephelometry
**Complete blood count**	
Thrombocytes (/nL)	Impedance measures according to Beckman-Coulter method
Erythrocytes (/pL)	Impedance measures according to Beckman-Coulter method
Leucocytes (/nL)	Impedance measures according to Beckman-Coulter method
Haemoglobin (g/L)	Impedance measures according to Beckman-Coulter method
Haematocrit (L/L)	Impedance measures according to Beckman-Coulter method
**Electrolyte panel**	
Potassium (mmol/L)	Flame photometry, spectrophotometry, direct or indirect ion selective electrode potentiometry
Sodium (mmol/L)	Flame photometry, spectrophotometry, direct or indirect ion selective electrode potentiometry
Magnesium (mmol/L)	Modified methylthymol blue complexometric procedure including Ba-EGTA
Phosphate (mmol/L)	Modified phosphomolybdate method including p-methylaminophenol sulfate and bisulfite
**Blood pressure**	
Systolic blood pressure (mmHg)	3 measurements with an oscillometric digital device (OMRON HEM-705CP). Average of 2nd and 3rd measurements
Diastolic blood pressure (mmHg)	3 measurements with an oscillometric digital device (OMRON HEM-705CP). Average of 2nd and 3rd measurements
Hypertension	Blood pressure ≥ 140/90 or use of antihypertensive medication given that participants were aware of having hypertension
**Liver parameters**	
GGT (U/L)	Modified IFCC method:including L-gamma-glutamyl-3-carboxy-4-nitranilide with glycylglycine
AST (U/L)	Modified IFCC method: P5Ü as activator and lactic acid dehydrogenase (LDH) to eliminate pyruvate interference
ALT (U/L)	Modified IFCC method: P5Ü as activator and hydroxymethyl aminomethane as buffer
Hepatic iron, right and left liver lobe (s^{-1})	MRI measurement (3 Tesla) by a single-voxel spectroscopy with a high-speed T2-corrected multi-echo (HISTO) technique

HFF, right and left liver lobe (%)	MRI measurement (3 Tesla) by a multiecho single-voxel 1H spectroscopy
**Further laboratory values**	
Alkaline phosphatase (U/l)	Bowers+McComb method: change in absorbance at 405 nm due to the formation of p-nitrophenol (p-NP) in the presence of the transphosphorylating buffer and AMP
CRP (mg/L)	CardioPhase hsCRP by particle enhanced immunonephelometry
Vitamin D (ng/mL)	Enhanced chemiluminescence immunoassay
Troponin T (pg/mL)	ECLIA Immunologic test
**Behavioral risk factors**	
Age (years)	Self-reported in standardized interview
Alcohol consumption (g/day)	Self-reported in standardized interview, calculated based on reported amount and type of beverages consumed, further information Bayerl et al. (35)
Smoking status	Self-reported in standardized interview, further information Bayerl et al.(35)
Pack years	calculated based on reported number of cigarettes smoked, further information Bayerl et al. (35)
Physically active	Self-reported in standardized interview, further information Rabel et al. (58)
**Medication intake**	
All medications[a]	Assessed by standardized interview. Participants were asked to bring packages of their medications from the last 7 days before the interview, further information Teuner et al. (59)

[a]please note that although the reference might not pertain to KORA FF4 but to one of the other KORA surveys, the described procedure was also applicable in FF4.

Table S2: SNPs included in the genetic risk score.

Marker of iron metabolism	SNP	Position	Gene	Alt Allele	MAF KORA	Weight, men	Weight, women	Ref
unsaturated iron binding capacity,<br>total iron binding capacity	rs2698530	2:64503895	MIR4433B	C	0.25	-0.165	-0.194	McLaren (60)
Ferritin	rs744653	2:190378750	SLC40A1	T	0.16	-0.736	0.596	Meidtner (27)
Ferritin	rs5742933	2:190649316	PMS1	C	0.20	0.495	0.480	Liao (61)
Transferrin	rs1799852	3:133475722	TF	T	0.11	-1.152	0.016	He (62)
Transferrin,<br>total iron binding capacity	rs3811647	3:133484029	TF	A	0.33	0.099	0.040	McLaren (60)<br>He (62)<br>Tayrac (29)
Ferritin,<br>Hepcidin,<br>Liver Iron	rs1799945	6:26091179	HFE	G	0.15	1.204	-0.273	Meidtner (27)<br>He (62)<br>Wilman (25)<br>Yuan (26)
Ferritin,<br>Hepcidin,<br>total iron binding capacity,<br>Liver Iron	rs1800562	6:26093141	HFE	A	0.05	1.876	3.269	McLaren (60)<br>Meidtner (27)<br>He (62)<br>Wilman (25)<br>Yuan (26)<br>Traglia (63)
Transferrin	rs4841132	8:9183596	PPP1R3B	G	0.08	0.162	1.133	Raffield (28)
Ferritin,<br>soluble transferrin receptor (sTfR)	rs236918	11:117091609	PCSK7	C	0.11	0.947	0.220	Meidtner (27)<br>Oexle (64)
Ferritin,<br>Hepcidin,<br>soluble transferrin receptor (sTfR),<br>Liver Iron	rs855791	22:37462936	TMPRSS6	G	0.43	-0.619	0.296	Meidtner (27)<br>He (62)<br>Wilman (25)<br>Yuan (26)<br>Traglia (63)<br>Oexle (64)<br>Nai (65)<br>Wainaina (66)
Hepcidin	rs4820268	22:37469591	TMPRSS6	A	0.45	-0.251	0.192	Wainaina (66)
Serum iron	rs1421312	22:37487810	TMPRSS6	G	0.38	-0.167	0.434	McLaren (67)
Ferritin	rs738409	22:44324727	PNPLA3	G	0.24	-0.027	0.374	Hotta (68)

MAF: minor allele frequency. Sex-specific weights were calculated from univariate regression of the respective SNP on outcome HIC.

Table S3: Reference intervals of laboratory values, with corresponding mean, minimum and maximum values in the study sample.

	Unit	Reference range	Ref	Study sample mean ± SD or median (IQR)	Study sample min	Study sample max
**Blood lipids**						
Total cholesterol	mg/dL	< 240	(69)	218.1 ± 36.7	133.0	347.0
HDL-C	mg/dL	> 40	(70)	62.1 ± 17.7	25.0	112.0
LDL-C	mg/dL	< 160	(70)	139.7 ± 33.3	50.0	230.0
Triglycerides	mg/dL	< 150	(70)	105.0 (76.9)	32.0	628.3
**Markers of glucose metabolism**						
Fasting glucose	mg/dL	70–110	(69)	103.2 ± 21.3	77.0	305.0
Fasting insulin	mU/mL	< 25	(69)	8.8 ± 7.4	0.1	54.0
HbA1c	%	< 5.7	(71)	5.54 ± 0.71	0.04	13.6
**Markers of renal function**						
Glomerular filtration rate	ml/min/1.73m^2	< 90	(72)	92.6 ± 16.8	29.5	122.8
Uric acid	mg/dL	m: 3.5 - 7.0<br>w: 2.5 - 6.5	(69)	m: 6.33 ± 1.32<br>w: 4.57 ± 1.11	2.15	11.76
Creatinine	mg/dL	m: 0.84 - 1.25<br>w: 0.66 - 1.09	(69)	m: 0.96 ± 0.13<br>w: 0.77 ± 0.12	0.55	1.26
Albumine	g/dL	3.5 - 5.3	(69)	4.35 ± 0.29	3.57	5.03
Cystatin C	mg/L	m: 0.54 - 0.94<br>w: 0.45 - 0.82	(69)	m: 0.89 ± 0.14<br>w: 0.85 ± 0.17	0.55	1.96
Urine albumine	mg/L	< 20	(73)	6.32 (8.85)	1.10	1070.0
Urine creatinine	g/L	0.3 - 2.5	(74)	1.60 ± 0.79	0.10	4.12
**Complete blood count**						
Haematocrit	L/L	m: 0.40 - 0.52<br>w: 0.35 - 0.47	(69)	m: 0.43 ± 0.03<br>w: 0.39 ± 0.03	0.29	0.50
Thrombocytes	/nL	150 - 450	(69)	230.9 ± 53.1	89.0	455.0
Erythrocytes	/pL	m: 4.40 - 5.90<br>w. 3.80 - 5.20	(69)	m: 4.87 ± 0.37<br>w: 4.45 ± 0.37	3.56	5.80
Leucocytes	/nL	4.30 - 10.0	(69)	5.65 (1.91)	2.50	15.02
Haemoglobin	g/L	m: 133 - 177<br>w: 117 - 157	(69)	m: 150.4 ± 10.1<br>w: 134.8 ± 9.8	94.0	179.0
**Electrolyte panel**						
Potassium	mmol/L	3.5 - 5.1	(69)	4.28 ± 0.28	2.92	6.13
Sodium	mmol/L	135 -145	(69)	139.0 (4.0)	121.0	145.0
Magnesium	mmol/L	0.65 - 1.05	(69)	0.86 ± 0.07	0.24	1.01
Phosphate	mmol/L	0.87 - 1.67	(69)	1.04 ± 0.15	0.53	1.49
**Liver parameters**						
GGT	U/L	m: < 60<br>w: < 40	(69)	m: 35.3 (33.9)<br>w: 19.6 (17.5)	7.0	348.0
AST	U/L	m: < 50<br>w: < 35	(70)	m: 24.5 (9.0)<br>w: 20.0 (8.0)	7.0	184.0
ALT	U/L	m: < 50<br>w: < 35	(70)	m: 31.0 (15.8)<br>w: 21.0 (12.0)	9.0	123.0

**Further laboratory values**						
Alkaline phosphatase	U/L	m: 40 - 130<br>w: 35 - 105	(69)	m: 65.9 ± 17.9<br>w: 67.6 ± 23.6	20.0	212.0
CRP	mg/L	< 5	(69)	1.12 (1.78)	0.16	25.0
Vitamin D	ng/mL	< 20	(75)	23.4 ± 11.6	4.2	64.0
hs-Troponin T	pg/mL	< 14	(76)	1.50 (3.82)	1.50	29.1

Table S4: Men: Results from unpenalized linear regression analyses from sensitivity analysis. β denotes the regression coefficient of the respective variable for outcome HIC. Adjusted R^2 denotes the variance of HIC explained. Presented are only variables with an inclusion frequency >20% in the variable selection procedure.

	**Adjustment**	**β**	**95% CI**	**p-value**	**adjusted R^2**
**Body composition**					
Waist circumference (cm)	age + HFF	-0.07	-0.14 ; 0	0.04	0.10
	age	0.02	-0.04 ; 0.07	0.49	0.01
**Blood lipids**					
(log) trigylcerides (mg/dL)	age + HFF	0.25	-0.94 ; 1.44	0.68	0.08
	age	1.11	-0.01 ; 2.22	0.05	0.03
**Markers of glucose metabolism**					
Fasting glucose (mg/dL)	age + HFF	-0.05	-0.07 ; -0.02	0.00	0.13
	age	-0.03	-0.06 ; 0	0.04	0.03
(log) 2-hour insulin (µU/mL)	age + HFF	0.06	-0.73 ; 0.86	0.88	0.12
	age	1.01	0.33 ; 1.69	0.00	0.05
2-hour glucose (mg/dL)	age + HFF	0.01	-0.01 ; 0.03	0.20	0.13
	age	0.02	0.01 ; 0.04	0.00	0.06
Prediabetes	age + HFF	0.92	-0.67 ; 2.5	0.25	0.11
	age	2.13	0.64 ; 3.63	0.01	0.05
**Complete blood count**					
Thrombocytes (/nL)	age + HFF	-0.01	-0.02 ; 0.01	0.38	0.08
	age	-0.01	-0.02 ; 0	0.20	0.02
Erythrocytes (/pL)	age + HFF	-1.35	-3.05 ; 0.35	0.12	0.09
	age	-1.03	-2.79 ; 0.73	0.25	0.02
**Electrolyte panel**					
(log) sodium (mmol/L)	age + HFF	4.63	-19.6 ; 28.86	0.71	0.08
	age	-2.89	-27.69 ; 21.92	0.82	0.01
Magnesium (mmol/L)	age + HFF	-2.40	-10.23 ; 5.43	0.55	0.08
	age	-2.22	-10.33 ; 5.9	0.59	0.01
**Blood pressure**					
Diastolic blood pressure (mmHg)	age + HFF	0.04	-0.02 ; 0.11	0.17	0.09
	age	0.08	0.02 ; 0.14	0.01	0.04
**Liver parameters**					
(log) hepatic fat fraction (%)	age	1.46	0.74 ; 2.19	0.00	0.08
**Behavioral risk factors**					
Alcohol consumption (g/day)	age + HFF	0.02	0 ; 0.05	0.04	0.10
	age	0.03	0.01 ; 0.05	0.01	0.04
**Medication intake**					
Calcium antagonists	age + HFF	-2.27	-4.77 ; 0.23	0.07	0.09
	age	-2.28	-4.87 ; 0.31	0.08	0.02

CI: confidence interval, HFF: hepatic fat fraction.

Table S5: Women: Results from unpenalized linear regression analyses from sensitivity analysis. β denotes the regression coefficient of the respective variable for outcome HIC. Adjusted R^2 denotes the variance of HIC explained. Presented are only variables with an inclusion frequency >20% in the variable selection procedure.

	Adjustment	β	95% CI	p-value	adjusted R^2
**Body composition**					
Height (cm)	age + HFF	-0.04	-0.13 ; 0.05	0.40	0.36
	age	-0.05	-0.15 ; 0.05	0.29	0.21
Visceral fat (L)	age + HFF	0.01	-0.12 ; 0.14	0.91	0.35
	age	0.81	0.5 ; 1.13	0.00	0.33
**Blood lipids**					
Total cholesterol (mg/dL)	age + HFF	0.01	-0.01 ; 0.02	0.45	0.36
	age	0.01	-0.01 ; 0.03	0.46	0.21
(log) trigylcerides (mg/dL)	age + HFF	-0.20	-1.8 ; 1.4	0.81	0.35
	age	1.70	0.14 ; 3.26	0.03	0.23
**Markers of glucose metabolism**					
Fasting glucose (mg/dL)	age + HFF	0.01	-0.02 ; 0.05	0.47	0.36
	age	0.05	0.01 ; 0.09	0.01	0.24
(log) fasting insulin (mU/mL)	age + HFF	-0.09	-1.13 ; 0.94	0.86	0.35
	age	1.35	0.43 ; 2.27	0.00	0.25
(log) 2-hour insulin (µU/mL)	age + HFF	-1.15	-2.21 ; -0.1	0.03	0.35
	age	0.56	-0.41 ; 1.54	0.25	0.20
**Markers of renal function**					
Uric acid (mg/dL)	age + HFF	-0.01	-0.6 ; 0.57	0.96	0.35
	age	0.73	0.18 ; 1.28	0.01	0.24
Urine creatinine (g/L)	age + HFF	-0.47	-1.14 ; 0.21	0.17	0.36
	age	-0.14	-0.89 ; 0.6	0.70	0.21
**Complete blood count**					
Thrombocytes (/nL)	age + HFF	0.01	-0.01 ; 0.02	0.34	0.36
	age	0.00	-0.01 ; 0.01	0.71	0.21
Erythrocytes (/pL)	age + HFF	-1.24	-3.02 ; 0.55	0.17	0.36
	age	-0.91	-2.9 ; 1.07	0.36	0.21
**Electrolyte panel**					
Potassium (mmol/L)	age + HFF	-2.74	-5.13 ; -0.34	0.03	0.38
	age	-2.44	-5.11 ; 0.22	0.07	0.22
(log) sodium (mmol/L)	age + HFF	-19.63	-45.67 ; 6.4	0.14	0.36
	age	-40.47	-67.31 ; -13.63	0.00	0.25
Phosphate (mmol/L)	age + HFF	2.62	-1.51 ; 6.76	0.21	0.36
	age	0.61	-3.94 ; 5.15	0.79	0.21
**Liver parameters**					
(log) hepatic fat fraction (%)	age	2.08	1.38 ; 2.79	0.00	0.36
**Further laboratory values**					
(log) CRP (mg/L)	age + HFF	-0.37	-0.92 ; 0.18	0.19	0.36
	age	0.17	-0.41 ; 0.75	0.57	0.21

Vitamin D (ng/mL)	age + HFF	-0.02	-0.07 ; 0.03	0.46	0.36
	age	-0.04	-0.1 ; 0.01	0.10	0.22
**Behavioral risk factors**					
Alcohol consumption (g/day)	age + HFF	0.05	0.02 ; 0.09	0.00	0.39
	age	0.07	0.03 ; 0.11	0.00	0.26
**Medication intake**					
Calcium antagonists	age + HFF	-2.24	-4.29 ; -0.19	0.03	0.37
	age	-2.35	-4.63 ; -0.07	0.04	0.23
Lipid-lowering agents	age + HFF	1.19	-0.67 ; 3.06	0.21	0.36
	age	1.89	-0.14 ; 3.93	0.07	0.22

CI: confidence interval, HFF: hepatic fat fraction.

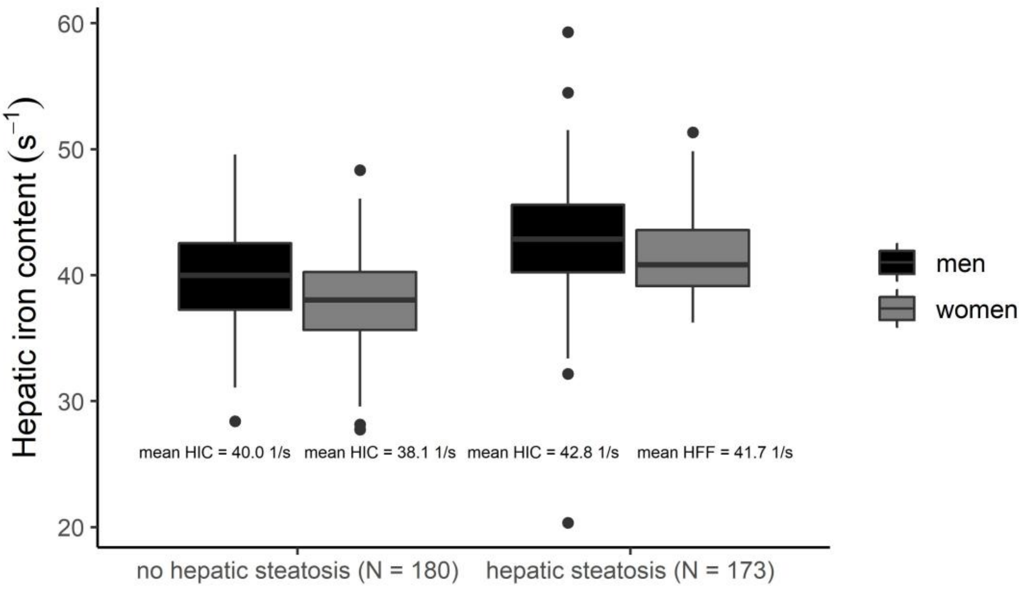

Figure S1: Boxplots of HIC according to hepatic steatosis status by sex. Cutoff for hepatic steatosis was HFF ≥ 5.6%, as defined by Schaapman et al. (55)

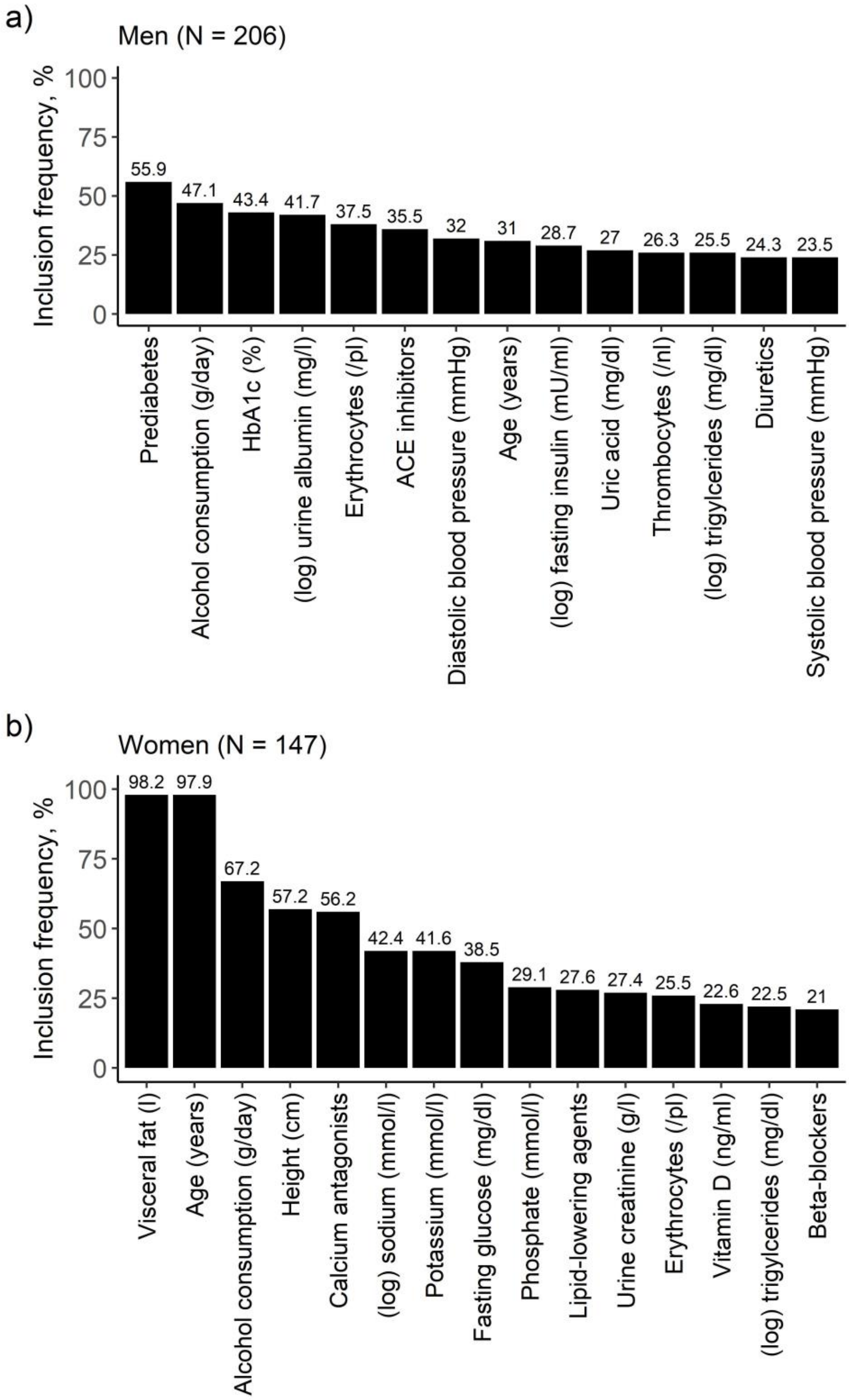

Figure S2: Bar diagrams of results from the model excluding HFF for a) men and b) women. Relevant variables were identified by variable selection through LASSO regression on 1000 bootstrap samples. On the y-axis: Inclusion frequency of the respective variable across 1000 bootstrap samples. Presented are only variables with an inclusion frequency > 20%.

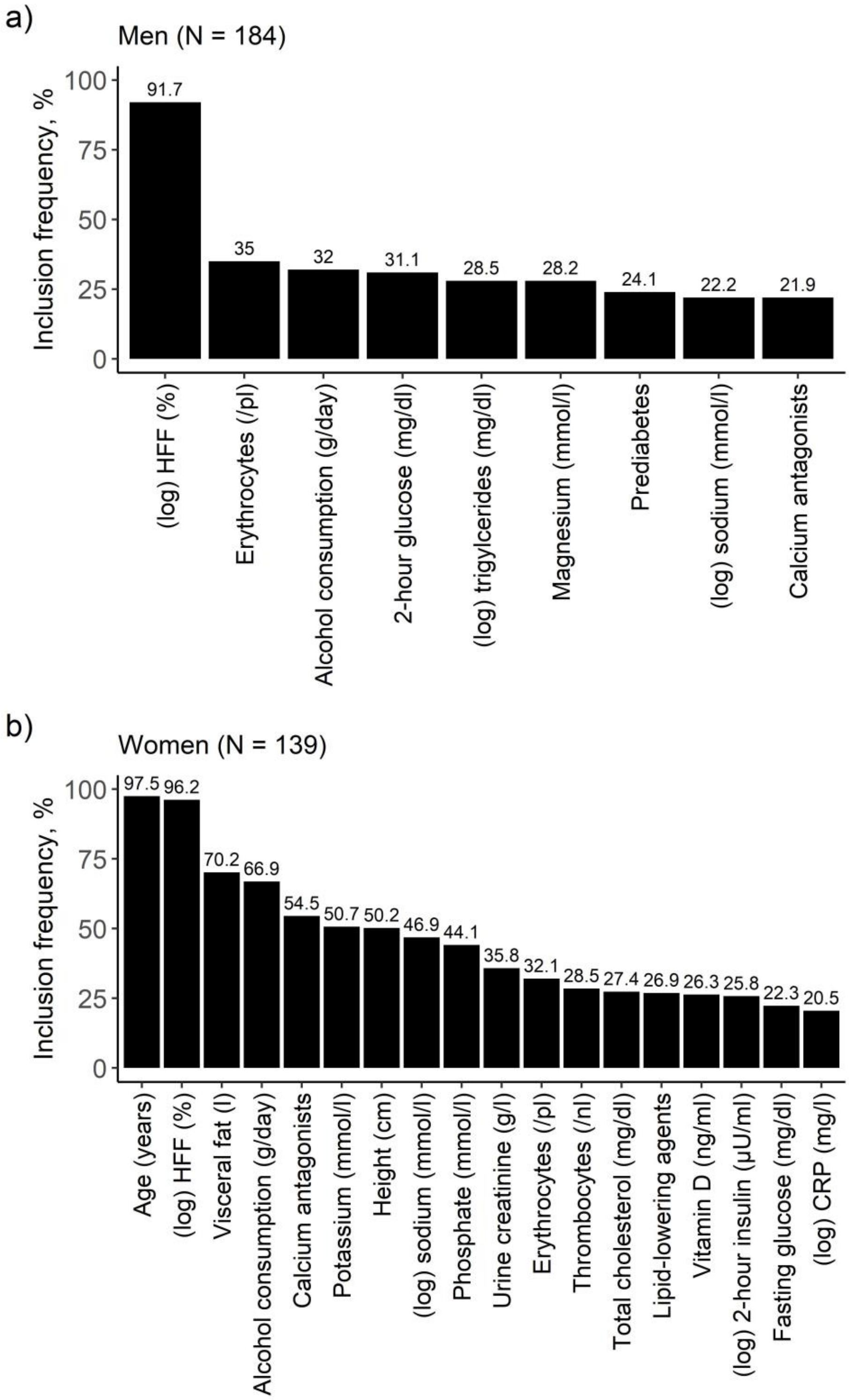

Figure S3: Bar diagrams of results from the sensitivity analysis including HFF for a) men and b) women. Relevant variables were identified by variable selection through LASSO regression on 1000 bootstrap samples. On the y-axis: Inclusion frequency of the respective variable across 1000 bootstrap samples. Presented are only variables with an inclusion frequency > 20%.

a)

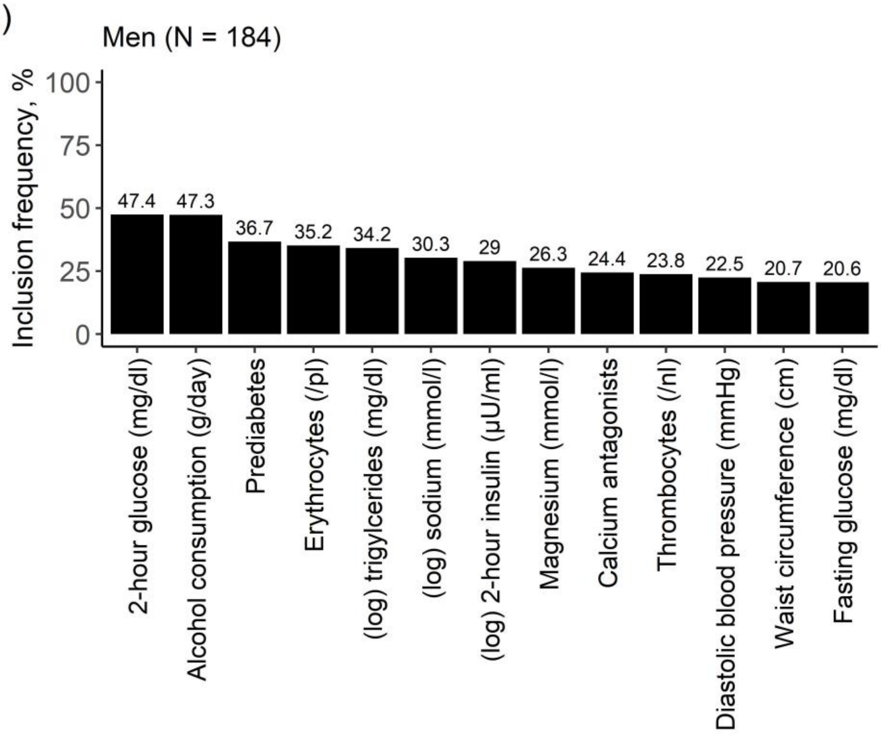

b)

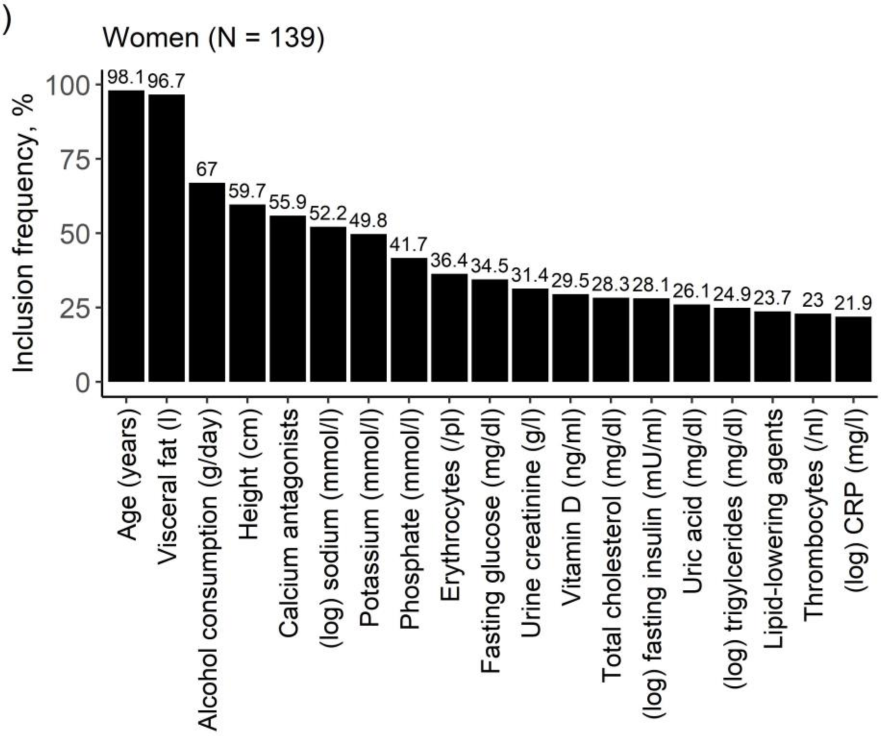

Figure S4: Bar diagrams of results from the sensitivity analysis excluding HFF for a) men and b) women. Relevant variables were identified by variable selection through LASSO regression on 1000 bootstrap samples. On the y-axis: Inclusion frequency of the respective variable across 1000 bootstrap samples. Presented are only variables with an inclusion frequency > 20%.
